# Exome sequencing of glioblastoma-derived cancer stem cells reveals rare clinically relevant frameshift deletion in MLLT1 gene

**DOI:** 10.1186/s12935-021-02419-4

**Published:** 2022-01-07

**Authors:** Hany E. Marei, Asmaa Althani, Nahla Afifi, Anwarul Hasan, Thomas Caceci, Armando Felsani, Giuseppe Tringali, Ingrid Cifola, Giacomo Pozzoli, Carlo Cenciarelli

**Affiliations:** 1 Department of Cytology and Histology, Faculty of Veterinary Medicine, Mansoura University, Mansoura, 35116 Egypt; 2 Biomedical Research Center, Qatar University, Doha, Qatar; 3 Qatar Biobank, Doha, Qatar; 4 Department of Mechanical and Industrial Engineering, College of Engineering, Qatar University, Doha, Qatar; 5 Biomedical Sciences, Virginia Maryland College of Veterinary Medicine, Blacksburg, VA USA; 6 Genomnia S.R.L, Via Ludovico Ariosto, Bresso, MI 20091 USA; 7 Institute of Pharmacology, Catholic University School of Medicine, Rome, Italy; 8 Institute for Biomedical Technologies (ITB), National Research Council (CNR), Milan, Italy; 9 Pharmacology Unit, Fondazione Policlinico A. Gemelli, IRCCS, Rome, Italy; 10 Institute of Translational Pharmacology (IFT), National Research Council (CNR), Via Fosso del Cavaliere, 100, 00133 Rome, Italy

**Keywords:** GBM, Exome, Sequencing, Cancer stem cells, Genetic variants

## Abstract

**Background:**

Glioblastoma multiforme (GBM) is a heterogeneous CNS neoplasm which causes significant morbidity and mortality. One reason for the poor prognostic outcome of GBM is attributed to the presence of cancer stem cells (CSC) which confer resistance against standard chemo- and radiotherapeutics modalities. Two types of GBM-associated CSC were isolated from the same patient: tumor core- (c-CSC) and peritumor tissue-derived cancer stem cells (p-CSC). Our experiments are focused on glioblastoma–IDH-wild type, and no disease-defining alterations were present in histone, BRAF or other genes.

**Methods:**

In the present study, potential differences in genetic variants between c-CSC versus p-CSC derived from four GBM patients were investigated with the aims of (1) comparing the exome sequences between all the c-CSC or p-CSC to identify the common variants; (2) identifying the variants affecting the function of genes known to be involved in cancer origin and development.

**Results:**

By comparative analyses, we identified common gene single nucleotide variants (SNV) in all GBM c-CSC and p-CSC, a potentially deleterious variant was a frameshift deletion at Gln461fs in the *MLLT1* gene, that was encountered only in p-CSC samples with different allelic frequency.

**Conclusions:**

We discovered a potentially harmful frameshift deletion at Gln461fs in the MLLT1 gene. Further investigation is required to confirm the presence of the identified mutations in patient tissue samples, as well as the significance of the frameshift mutation in the MLLT1 gene on GBM biology and response to therapy based on genomic functional experiments.

**Supplementary Information:**

The online version contains supplementary material available at 10.1186/s12935-021-02419-4.

## Introduction

Since it is one of the most aggressive and recurrent brain tumors, glioblastoma multiforme (GBM) poses a great health problem. The recurrent nature of GBM is principally attributed to the presence of a group of tumor-initiating cells, cancer stem cells (CSC); these are thought to play decisive roles in GBM’s resistance to radio- and chemotherapy, resulting in a typically fatal tumor recurrence ~ 7 mo after diagnosis [1].

In our previous studies, we identified two types of CSC within the tumor core (c-CSC), and in the peritumor tissue of GBM (p-CSC) [2–4]. The genetic makeup and driver mutations of the primary and recurrent GBM primary tumor cells and their associated CSC is not clarified yet. The presence of a heterogeneous population of cancer cell clones within GBM necessitates a thorough understanding of potential genetic variants and their interrelation to each other. Elucidation of the clonal structure and underlying genetic variants in GBM is thus crucial to develop targeted therapies for lethal cancer, such as GBM.

As the name implies, the term ''multiforme'' indicates the high degree of heterogeneity not only in the histopathological features of glioblastoma but also in its genetic mutational load. Previous studies have classified GBM mutations into two main types: clonal and subclonal ones. The clonal mutations are identified in all tumor cells before the process of transformation, while the subclonal mutations are present only in a subset of tumor cells; and they occur later in tumor growth [5]. Whether or not the GBM CSC are clonal or subclonal mutations is not clear yet, and the role of GBM CSC in either the initiation and/or maintenance of GBM growth is still in need of further investigation.

Three core signaling pathways, namely p53, Rb, and receptor tyrosine kinase (RTK)/Ras/phosphoinositide 3-kinase (PI3K) have been previously implicated as playing a main role in the initiation of GBM growth [6]. Alteration in the core molecular pathways is thought to be coordinated and clearly imprinted in different molecular subtypes of GBM. This is clearly reflected not only inpatient sensitivity to different therapeutic modalities but also it appears to have pronounced effects on the clinical outcome [7].

Previous genomic studies of GBM reveals the presence of 21,540 somatic mutations in 71 GBM-relevant genes of which 20,448 were single-nucleotide variants (SNVs), 39 were dinucleotide mutations, and 1,153 were small insertion and deletion (indel) mutations. The SNVs mutations included 5379 silent, 3901 missense, 831 nonsense, 360 splice-site, and 760 mutations resulting in a frameshift [8]. Several genes were identified as significantly mutated genes in GBM namely PTEN, TP53, EGFR, PIK3CA, PIK3R1, NF1, RB1, IDH1, PDGFRA, LZTR1, SPTA1, ATRX, GABRA6, KEL, BRAF V600E, H3.3 histones, EGFR, MET, CDK6), CDK4, MDM2, PDGFRA, SOX2, MYCN, CCND1, CCNE2, PARK2, QKI, TGFbR2, LRP1B, NPAS3, LSAMP, SMYD3, EGFR, CPM, PRIM2, FAM65B, PPM1H, RBM25, HOMER2, EGFRvIII, PDGFRA, p53 pathway (MDM2, MDM4, and TP53), the Rb pathway (CDK4, CDK6, CCND2, CDKN2A/B, and RB1), PI3K pathway (PIK3CA, PIK3R1, PTEN, EGFR, PDGFRA, and NF1), IDH1, ATRX, TERT [8].

In our previous studies, Notch inhibition significantly impaired cell growth of c-CSC compared to p-CSC pools, with no effects observed in cell cycle distribution, apoptosis, and cell invasion assays. Moreover, there was simultaneous targeting of EGFR and PDGFR, which would be beneficial in the treatment of GBM [3]. In another study, the newly discovered PDGFRα/Stat3/Rb1 regulatory axis may represent a potential therapeutic target for GBM treatment [4]. Moreover, Hes1 seems to be a favorite target but not sufficient itself to target GBM efficaciously; therefore we suggested that any potential pharmacological intervention should provide for the use of anti-Stat3/5 drugs either alone or in combination regimen [2].

The complex interrelated molecular pathway that governs the tumorigenic transformation of GBM c- and p-CSC has prompted us to perform a systemic genomic study using whole-exome sequence (WES), to explore potential differences in the global genetic variants between GBM-associated c-CSC and p-CSC. In this regard, four pairs of c-CSC) or p-CSC tissue-derived cancer stem cells were isolated from 4 different patients with the aims of (1) obtaining the complete sequence of the exome of the 4 pairs of GBM cancer stem cell lines, (2) obtaining, for each CSC, the list of the variants respect to the reference genome, (3) comparing the exome sequences between each pair of c-CSC and p-CSC, identifying the different and common variants, and (4) identifying the variants that affect the functionality of genes known to be involved in cancer origin and development. Our experiments are focused on glioblastoma–IDH-wild type, and no disease-defining alterations were present in histone, BRAF or other genes.

By comparative analysis, we identified common gene single nucleotide variants (SNV) in all GBM c-CSC, and p-CSC in the following genes: *TP73, PDE4DIP, FN1, KMT2C, MUC6, CREB3L1, GSE1, APC2, and MUC16*. A potentially deleterious variant was a frameshift deletion at Gln461fs in *MLLT1* gene, which was encountered only in p-CSC. Our study supports the hypothesis that the varied genetic composition between the GBM-associated c-CSC and p-CSC may be involved in the different therapeutic responses or the recurrent nature of GBM. Moreover, the design of specific targeted therapeutic strategies against the most critical/penetrant variants affecting the functionality of GBM should be directed to the genetic alteration associated with both the primary GBM tumor cells and GBM-associated CSC.

## Materials and methods

### Ethical statement

Procedures for collection of adult human GBM CSC were approved by the Ethical Committee of the Catholic University of Rome as reported previously [9]. Informed consent was obtained, and all patients were fully aware of the aims and scope of this work. The ethical principles of the declaration of Helsinki were strictly followed.

### Cell culture of human Glioblastoma cancer stem cells

The GBM cells were identified based on their histology, and EGFR wt or Variant III by PCR. They were negative for EGFR VIII except GBM CSC1. Moreover, whole exome analysis of GBM CSCs did not detect EGFRvIII. We found EGFRvIII only in GBM c-CSC1 subsequently by RT-PCR as published previously 3. The GBM cells used in the present study were not tested for IDH1/2 gene mutations because those are rarely found in primary GBM. Moreover, IDH1/2 whole exome analysis of GBM CSC did not reveal any relevant gene mutations.

Glioblastoma (GBM) tissue specimens were obtained from either the very core of the tumor (avoiding necrotic tissue) or from at least 2 cm away from it. Using the neurosphere protocol which isolates GBM cancer stem cells (CSC), stable GBM cell cultures were established from the periphery (p-CSC) and the core (c-CSC) using specimens derived from the same patient. Four pairs of core- (c-CSC) or peritumor- (p-CSC) tissue-derived cancer stem cells were isolated from 4 different patients. Unfortunately, the blood genomic DNA of the 4 patients is not available now. The procedure is aimed at sequencing only the transcribed and coding regions of the genome, which represents about 1/60 of the total in the human being. Since these regions are much more highly studied and annotated than others, genetic analysis and the interpretation of the sequencing data is simplified. Therefore, investigators can perform genetic studies with higher and statistically more relevant numbers of samples.

We used the same clinical materials reported in our previous papers [9, 10]. In brief, the CSC cells were retrieved from adult patients affected by GBM and undergoing craniotomy at the Institute of Neurosurgery, Catholic University-School of Medicine of Rome, Italy. Dissociated cells were cultured in the presence of human recombinant EGF (20 ng/ml; PeproTech, Rocky Hill, NJ), human recombinant bFGF (10 ng/ml; PeproTech), in DMEM/F12 (1:1) serum-free medium (Invitrogen, Carlsbad, CA) containing l glutamine 2 mM, glucose 0.6%, putrescine 9.6 µg/ml, progesterone 0.025 mg/ml, sodium selenite 5.2 ng/ml, insulin 0.025 mg/ml, apo transferrin sodium salt 0.1 mg/ml, sodium bicarbonate 3 mM, Hepes 5 mM, BSA 4 mg/ml, heparin 4 µg/ml (all purchased from Sigma-Aldrich). Floating neurospheres were dissociated with Accutase at 37 °C (Merck-Millipore). In some cases, neurospheres were passaged up to passage P30 and the experiments were performed between P14 and P30. The GBM-derived c-CSC cultures (# 1, 2, 3, 4) are primary cells with a limited life span. First, following 30 passages, the proliferation rate of the cells is increasingly reduced ending up to cell cycle arrest. Second, small tumor spheres display a necrotic phenotype. The experiments were performed with mycoplasma-free cells.

### DNA extraction

Genomic DNA was extracted from cell pellets using the Qiagen DNeasy Blood & Tissue Kit, according to the manufacturer’s instructions, and including the RNAse A optional treatment. DNA amounts were quantified by Quant-iT™ DNA Assay Kit, High Sensitivity. Eight barcoded exome libraries were constructed starting with 80 ng of genomic DNA using the Ion AmpliSeq™ Exome RDY S5 Kit (Thermo Fisher). The quality and amount of the libraries were checked by Bioanalyzer. An equimolecular pool of the barcoded libraries was made and used by the Ion Chef to prepare the templated beads that were then sequenced on two ION540 chips using the Ion S5™ System (Thermo Fisher).

Sequencing reads were mapped to the human reference genome (genome build hg19) using the software Torrent Suite (5.4.0). The mapping rate was above 99%. An average of 21 million mapped reads was obtained per sample, corresponding to an exome coverage of about 70 times. The Bam files were then analyzed for variants with the Exome Single Sample Somatic workflow on Ion Reporter (release 5.6).

### Whole exome sequencing

The whole-exome procedure includes the preparation of a genomic DNA fragment library coupled with exon region enrichment. The enrichment system used is the Agilent SureSelect™ Target Enrichment kit. The Agilent SureSelect™ kit is continuously updated, as far as exome coverage and annotation are concerned, and version 5 (presently in use) covers 50 Mb of exons of 21522 genes. The SureSelect™ Target Enrichment workflow is a solution-based system which is based on the capture of the complementary DNA regions of the library through 120 nt biotinylated cRNA baits covering the target exomic regions. RNA–DNA hybrids are subsequently enriched out of the fragment library using streptavidin magnetic beads.

To identify tumor-specific variants and p- and c- specific, each c-CSC and p-CSC sample all the variants with the reference genome were reported and annotated concerning dbSNP and COSMIC. All the annotated functional variants common to all c-CSC samples and to all p-CSC samples separately were identified. These ‘putative tumor variants’ were then compared between the two groups of samples, highlighting the similarities and differences. Identical functional damaged variants between p- and c-samples could be tumor-specific. Similar functional variants different between the two groups could be instead p- or c-specific.

Alignments concerning the reference genome in the binary format “. bam" and relative indexes “. bai", were delivered on a portable HD or a set of DVDs, depending on the final size of the data. Metrics of sequence coverage for exons and enrichment were relative to the capture kit used. For the genome regions enclosed in the capture kit, there was the identification of SNPs and small insertions—deletions (max 20 nt). Mapping of the variants concerning UCSC or Ensembl gene annotations. There was a comparison to known variants already included in dbSNP (variant classifications as NEW or KNOWN). Annotations of the characteristics of the sequencing included coverage of the whole sequence and wild type variant alleles relative to the reference genome, giving values of alignment quality. Features of SNPs were identified as belonging to dbSNP or COSMIC. Generation of files of variants (SNPs and INDELs separately) were in tabular text format and standard ".vcf". Functional annotation of the identified variants, including the prediction of the possible functional effects with SIFT and Polyphen prediction tools, and a list of information such as type of variants, zygosity, reference and variant alleles, dbSNP ID, EnsEMBL Gene ID, Gene Name, Protein Effect, cDNA position, CDS position, predicted effect, coverage, and percent of variant reads.

### Mapping and variant calling

Sequencing reads were mapped to the human reference genome (genome build hg19) using the Torrent Suite (5.4.0). The Bam files from the two chips were merged using the Combine Alignments TS utility and finally analyzed with the Exome Single Sample Somatic workflow on Ion Reporter (release 5.6).

### Sample identity-check

To understand whether the center and periphery samples came from the same patients, we calculated the number of variants shared between center and periphery in each sample pair. Samples coming from the same patient should have a high number of variants shared between center and periphery, while samples coming from different subjects should have a lower number of shared variants.

### Selection of interesting variants

For each patient, we identified: (1) Variants detected both in the tumor center and in the tumor periphery, (2) Variants detected only in the tumor center, and (3) Variants detected only in the tumor periphery. The results from all patients were then analyzed collectively to identify: (1) Variants detected in the tumor center in all patients, (2) Variants detected in the tumor periphery in all patients, (3) Variants detected in all patients, both in the center and the periphery, (4) Variants detected in all patients only in the tumor center, and (5) Variants detected in all patients only in the tumor periphery. Moreover, for all these categories, we highlighted the potentially pathogenic variants: Variants with population frequency ≤ 1%, variants with a strong impact on the amino acid sequence (i.e.: frameshift indels; variants introducing or deleting stop codons; variants in splice sites; missense variants predicted to be potentially damaging for protein functionality according to SIFT and/or PolyPhen tools), and variants in known oncogenes. For this annotation, we used the oncogene list available on http://www.bushmanlab.org/links/genelists (last updated February 2017).

To perform the variant prioritization and select the potentially pathogenic mutations we used the Excel filters on the following columns: (1) COSMIC cancers: by selecting nonempty cells we can view the variants associated with several cancers according to the COSMIC database, (2) Rare Mutation: the “Y” cells highlight the rare mutations, i.e. those with a population frequency ≤ 1%, (3) High Impact: by selecting the cells with “Y” we can highlight the variants with a strong impact on the amino acid sequence (i.e.: frameshift indels; variants introducing or deleting stop codons; variants in splice sites; missense variants predicted to be potentially damaging for protein functionality according to SIFT and/or PolyPhen tools), (3) High Quality: by selecting the cells with "Y" we can view the variants with a PHRED quality score greater than 40, thus high-quality calls, and (4) Oncogenes: by selecting the non-empty cells we can view the variants affecting known oncogenes (Table 1).

## Results

### General description of samples

We evaluated 4 pairs (one c- and one p-GBM CSC) from four GBM patients; in the 4 patients, we were able to analyze both the c- and p-CSC (Additional file 1: Table S1).

### Mapping statistics (hg19)

The total reads length for all samples ranged between ~ 18,000 to 29,000 reads, and the mapping reads ranged between 12,000 to 29,000 reads. This gave an approximate mapping rate between 75 to 99.66%, and an on-target mapping of 87 to 96% (Table 2). After filtering and visual inspection, the sequencing results revealed 1742, 2181, 2345, 2284 high confidence somatic mutations in the CSC1, CSC2, CSC3, and CSC4 samples, and 2132, 2307, 1990, and 4004 ones in p-CSC1, CSC2, CSC3, and CSC4, respectively (see “Materials and methods” section).

**Table 1:** A list of analyzed samples

Sample	Internal ID	Barcode	Type	Organism
c-CSC1	1	1	Core glioblastoma cancer stem cells	Human
p-CSC1	2	2	Peritumor glioblastoma cancer stem cells	Human
c-CSC2	3	3	Core glioblastoma cancer stem cells	Human
p-CSC2	4	4	Peritumor glioblastoma cancer stem cells	Human
c-CSC3	5	5	Core glioblastoma cancer stem cells	Human
p-CSC3	6	6	Peritumor glioblastoma cancer stem cells	Human
c-CSC4	7	7	Core glioblastoma cancer stem cells	Human
p-CSC4	8	8	Peritumor glioblastoma cancer stem cells	Human

**Table 2:** Mapping statistics

Sample	Total reads	Mapped reads	Mapping rate (%)	On target (%)
c-CSC1	12,563,290	12,520,354	99.66%	94.76%
p-CSC1	21,381,239	21,313,206	99.68%	96.03%
c-CSC2	23,975,039	23,897,330	99.68%	95.27%
p-CSC2	23,223,428	23,154,328	99.70%	95.68%
c-CSC3	29,635,293	29,533,507	99.66%	95.32%
p-CSC3	20,265,457	20,192,375	99.64%	95.85%
c-CSC4	23,921,320	23,823,634	99.59%	95.27%
p-CSC4	18,706,321	14,175,267	75.78%	87.85%

### Sample identity-check

**Fig. 1:**
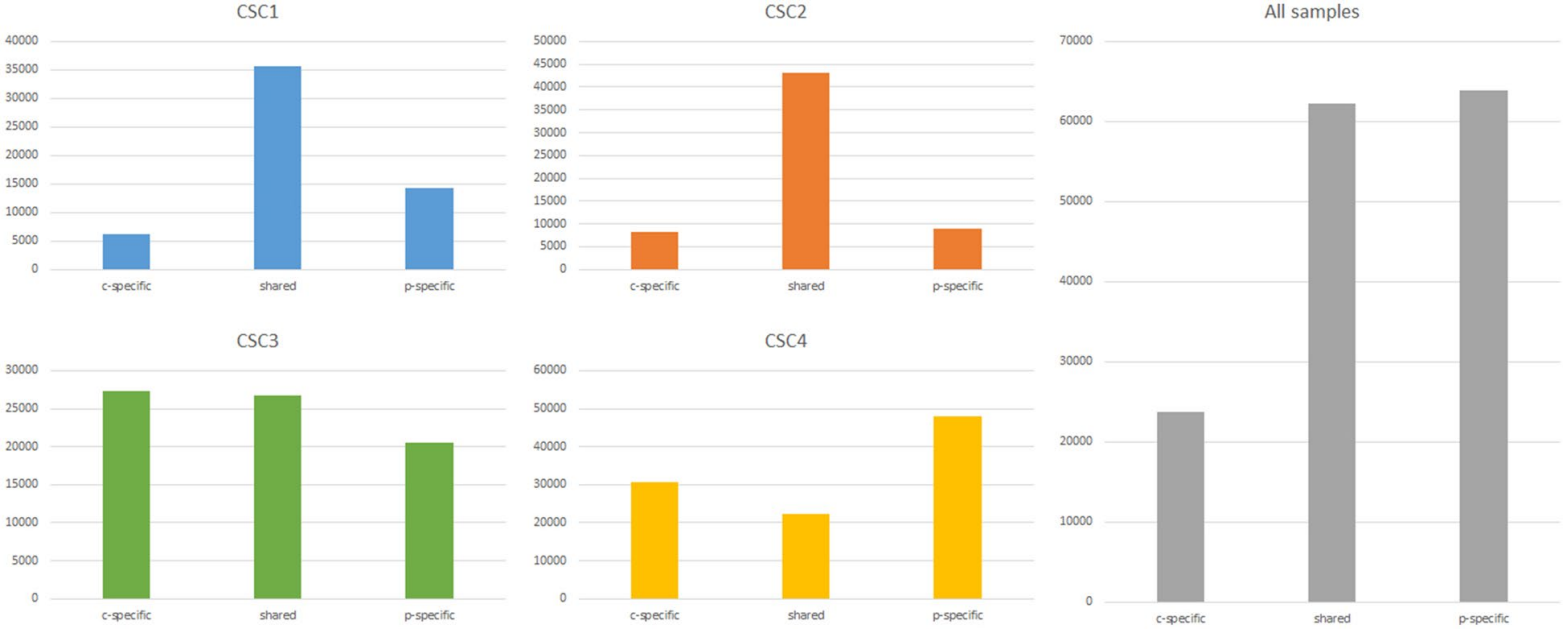
Shared and specific variants identified in the 4 cell lines used

**Fig. 2:**
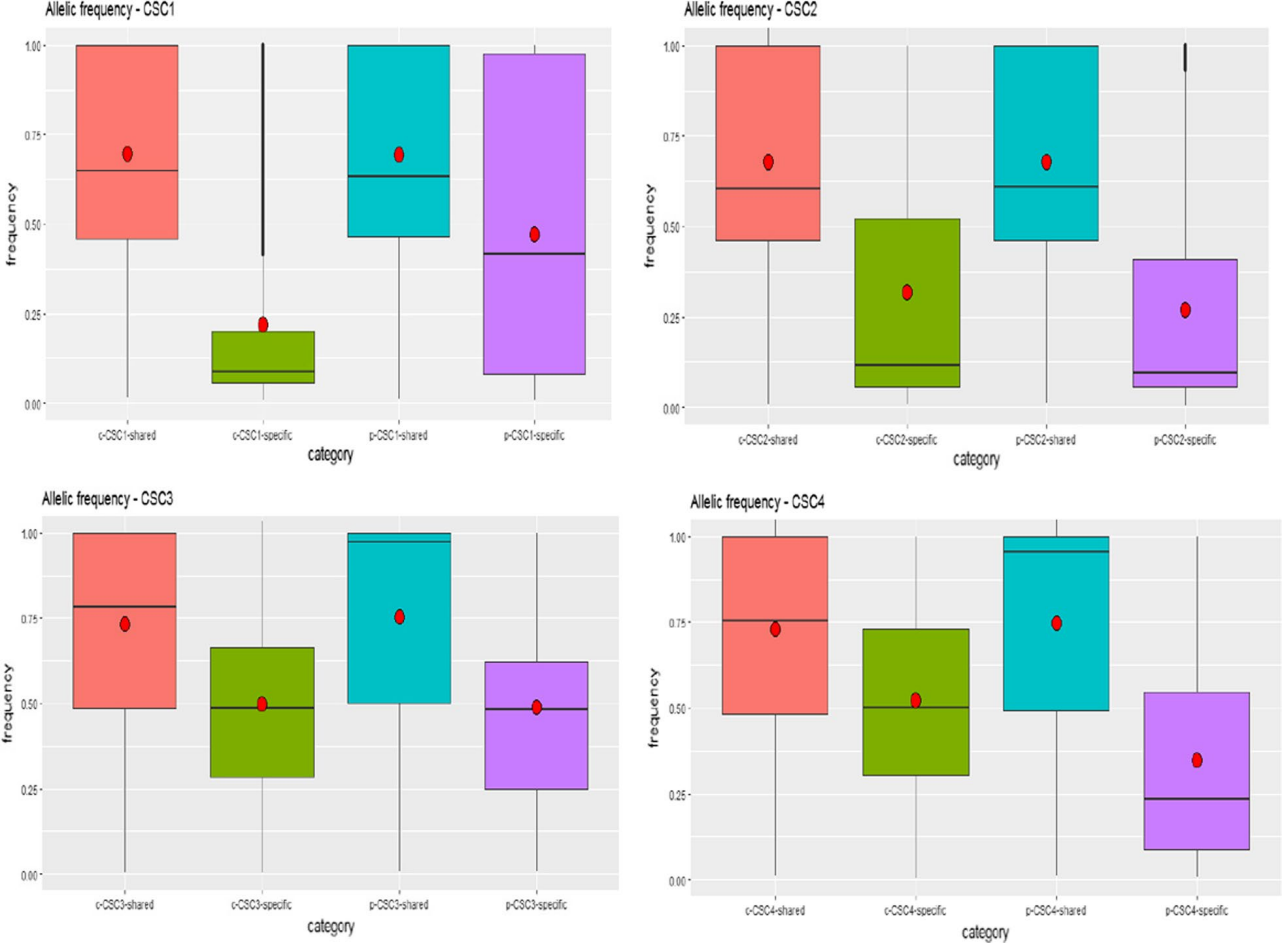
Allelic frequencies of the used 4 cell lines (c- vs p-CSC)

We tested potential matches between the center and the periphery samples for both CSC1 and CSC2 patients. In fact, in these patients, most variants were shared between the center and the periphery sample. On the contrary, for CSC3 and CSC4 we could not confirm a tight match between center and periphery: in these cases, a lower number of variants were shared between the samples, and most variants were center-specific or periphery-specific (Figs. 1 and 2, Additional file 2: Table S2).

### Selection of common variants in the center and periphery GBM CSCs

By selecting the rare variants of high quality affecting known oncogenes with a strong impact on the coding sequence, the following candidates’ genes and associated variants look particularly interesting, when they are detected in all patients, both in the center and the periphery sample. The ten mutated genes that were identified were TP73, PDE4DIP, FN1, KMT2C, MUC6, CREB3L1, GSE1, APC2, and MUC16 (Table 3). One indel variant in MLLT1 genes was characteristic only in the periphery samples (Table 4). As for the variants detected in all patients only in the center samples, no rare high-quality variants were found affecting known oncogenes with a strong impact on the coding sequence. However, a frameshift deletion (p.Tyr118fs) was found in gene DLX6/DLX6-AS1, with unknown population frequency.

**Table 3:** Variants detected in all patients, both in the centre and the periphery sample

	Reference	Variant	Type	Zygosity	Amino acid change	Oncogene
chr1:3,644,245	G	T	SNV	HET	p.Gly299Val	TP73
chr1:144,866,643	G	A	SNV	HET	p.Arg1867Cys	PDE4DIP
chr1:144,879,375	T	C	SNV	HET	p.Lys1359Glu	PDE4DIP
chr1:144,882,823	C	T	SNV	HET	p.Ala1066Thr	PDE4DIP
chr1:144,917,841	T	C	SNV	HET	p.His482Arg	PDE4DIP
chr1:144,918,957	T	A	SNV	HET	p.Glu410Val	PDE4DIP
chr1:144,922,583	G	A	SNV	HET	p.Ser275Leu	PDE4DIP
chr1:144,994,658	C	A	SNV	HET	p.Arg25Leu	PDE4DIP
chr2:216,272,900	T	G	SNV	**HOM**	p.Thr817Pro	FN1
chr7:151,945,071	G	GT	INDEL	HET	p.Tyr816Ter	KMT2C
chr11:1,017,183	G	T	SNV	HET	p.Pro1873Gln	MUC6
chr11:1,017,220	T	C	SNV	HET	p.Thr1861Ala	MUC6
chr11:1,017,325	A	C	SNV	HET	p.Tyr1826Asp	MUC6
chr11:1,017,337	TC	CA	MNV	HET	p.Gln1821_Thr1822delinsHisAla	MUC6
chr11:46,342,081	TG	T	INDEL	**HOM**	splicesite_3	CREB3L1
chr11:46,342,259	A	AG	INDEL	**HOM**	splicesite_5	CREB3L1
chr16:85,667,696	G	A	SNV	HET	p.Ala62Thr	GSE1
chr19:1,457,111	C	A	SNV	HET	p.Pro359Gln	APC2
chr19:9,087,615	T	A	SNV	**HOM**	p.Lys1400Asn	MUC16

**Table 4:** Variants detected in all patients, only in the periphery sample

Position	Reference	Variant	Type	Zygosity	Amino acid change	Oncogene
chr19:6,213,974	CTG	CT	INDEL	HET	p.Gln461fs	MLLT1

### Selection of center-specific or periphery-specific variants

The following candidate's genes and associated variants were detected in all patients in the center of the samples. The twelve center-specific variants genes identified were: TRAF3IP3, PATJ (three variants), MIR1237/RPS6KA4, BISPR/BST2, FOSB, HPSE, AKAP7, CYP21A1P/ CYP21A2, EZH2, DLX6/DLX6-AS1 (Table 3). Most of these variants are synonymous or unknown substitutions. Only PATJ variant, p.Gly1178Ser, is a missense substitution in exon 6, and with allelic frequency 1 (Additional file 3: Table S3) in all center samples and described in clear cell renal cell carcinoma (ccRCC) and acute_myeloid_leukaemia. A decrease of PATJ in ccRCC was associated with the male advanced tumor, and poorer survival, suggesting that PATJ may be a useful prognostic biomarker and therapeutic target for ccRCC [11]. Another clinically relevant variant was found in DLX6/DLX6-AS1, p.Tyr118fs, in exon 1, introducing a frameshift mutation. LncRNA DLX6-AS1 was both high-expressed in the glioma cells and tissue, and the overexpression was clinically correlated with the poor outcome of glioma patients [12].

The following candidate's genes and associated variants were detected in all patients in the periphery of the samples. The eleven periphery-specific variants genes identified were: DNAH14, PSEN2, ABCB10, COL13A1, POLE2, MYH1/MYHAS, MLLT1, FAM228B, ITSN2, EIF2A, HMGA1 (Additional file 3: Table S3). Most of the variants are synonymous and unknown. DNAH14 genetic rare variation introduces a missense amino acid substitution in p.Leu4096Pro, in exon 77. Genetic variation in DNAH14 rs3105571 has been described and is significantly associated with pathologic complete response to neoadjuvant chemoradiotherapy in locally advanced rectal cancer [13]. Another clinically relevant frameshift deletion was found in MLLT1 gene, p.Gln461fs, in exon 6. MLLT1/ENL gene plays pivotal roles in the regulation of chromatin remodeling and gene expression of many important proto-oncogenes, such as Myc, Hox genes, via histone acetylation [14] (Additional file 3: Table S3).

### Exploration of TCGA data base for potential link between GBM and the identified frameshift mutation in the MLLT1 gene

To further highlight the role of the identified frameshift mutation in the MLLT1 gene, we looked into the TCGA data to learn more about the potential role of the frameshift deletion at Gln461fs in the MLLT1 gene and its involvement in GBM tumorigenicity. There are three cases in the TCGA-GBM with MLLT1 mutations, but they are of a different type than our Gln461fs. Furthermore, they have effects on different regions and amino acids than the one we identified. The same frameshift mutation that we found in this study (indicated in TCGA as AA change Q461Rfs*46 Gln- > Arg, given by chr19:g.6213965delG) has been found in four other tumours: three colon adenocarcinomas and one stomach adenocarcinoma. In all cases, the mutation has an impact on protein functionality and is classified as "High" (i.e., highly deleterious for protein functionality). Notably, with four cases, this frameshift mutation is considered to be one of the two most common mutations in the coding portion of MLLT1 that have been reported in TCGA, the other one being a missense S305L mutation, which has also been reported in four TCGA cases [15].

## Discussion

In this study, we used exome sequencing to provide a list of somatic/rare alterations associated with GBM. Specifically, we are interested in highlighting potential variants associated with oncogenic genes both in c- and p GBM CSC. Linking genomic data to clinical information might provide new opportunities to decipher genomics-based biomarkers, and to generate novel hypotheses which might help to highlight novel disease-related mechanisms. Our experiments are focused on glioblastoma–IDH-wild type, and no disease-defining alterations were present in histone, BRAF or other genes.

In the present study, SNV was identified in the oncogene *TP73* both in C- and p-GBM CSC. *TP73-AS1* constitutes a clinically relevant lncRNA in GBM. Significant overexpression of *TP73-AS1* was previously identified in primary GBM samples and comprises a prognostic biomarker in glioma and GBM with high levels of expression, identifying patients with particularly poor prognoses. *TP73-AS1* promotes TMZ resistance in GBM CSC and is linked to regulation of the expression of metabolism-related genes and ALDH1A1, a protein known to be expressed in cancer stem cell markers and which protects GBM CSC from TMZ treatment [16].

Several SN heterozygous variants in PDE4DIP have been detected both in c- and p-GBM CSC. In all cases, the genomic alterations were associated with amino acid changes such as: p.Arg1867Cys (chr1:144866643), p.Lys1359Glu (chr1:144879375), p.Ala1066Thr (chr1:144882823), p.His482Arg (chr1:144917841), p.Glu410Val (chr1:144918957), and p.Ser275Leu (chr1:144922583) (Table 3). The role of *PDE4DIP* in GBM was previously reported: it has been demonstrated that this gene is down-regulated in glioma cell lines treated with dB-cAMP. a hat reduces the invasiveness, proliferation, and migratory properties of glioma cells and increases the survival of glioma cell lines compared to untreated cell lines [17, 18].

A homozygous SNV was detected in *FN1* gene in the examined in c- and p GBM CSC. This variant was detected at chr2:216272900 where a homozygous T-G transition that was associated with p.Thr817Pro was identified. Activations of MYC, NFE2L2, FN1, and TGFβ1 and inhibition of TP53 in GBM were previously demonstrated by Halla et al. [19]. *FN1* is upregulated by *TWIST1*, which is known to promote epithelial-mesenchymal transition and/or GBM invasion [20, 21]. Furthermore, *FN1* is associated with glioblastoma recurrence and can be regarded as a target for antiangiogenic therapy [22]. COL1A1 and FN1 are associated with migration, invasion, angiogenesis, recurrence, and OS in GBM patients. Thus, these genes may serve important roles in the tumorigeneses of GBM [20].

The present study revealed the presence of heterozygous indel in the KMT2C gene. This indel was detected at chr7:151945071 position, and it was associated with p.Tyr816Ter. KMT2C in a GBM is rare; this mutation occurs in only about 4% of GBMs. However, all sorts of other cancers show this mutation. Alterations of EZH2, KMT2C, and CHD4 at the genetic or protein level could perturb an epigenetic program, leading to malignant transformation in glioma [23].

Four heterozygous SNV were detected in *MUC6* gene at chr11:1017183, chr11:1017220, chr11:1017325, and chr11:1017337. These variants were associated with p.Pro1873Gln, p.Thr1861Ala, p.Tyr1826Asp, and.Gln1821_Thr1822delinsHisAla, respectively. The MUC6 gene encodes gastric mucin, a secreted glycoprotein that plays an essential role in epithelial cytoprotection from acid, proteases, pathogenic microorganisms, and mechanical trauma in the gastrointestinal tract [24]. The susceptibility to gastric cancer may be related to variation in MUC6 gene expression [25].

Two homozygous indels were detected in CREB3L1 gene at chr11:46342081, and chr11:46342259. Normal and tumor tissues with similar CREB3L1 expression include ESCA esophageal cancer, GBM glioblastoma multiforme, HNSC head, and neck squamous cell carcinoma, LUAD lung adenocarcinoma, SARC sarcoma, THCA papillary thyroid carcinoma, THYM thymoma, UCEC uterine corpus endometrial carcinoma [26]. CREB3L1 is a member of the CREB/ATF family of transcription factors and functions as a transducer of the unfolded protein response (UPR) [27]. A large fraction of proteins synthesized in the cell undergoes folding and post-translational modification in the endoplasmic reticulum before being released to perform their desired function. This process can be disrupted by endoplasmic reticulum stress resulting from hypoxia, glucose or nutrient depletion, change in calcium homeostasis, or expression of mutant or misfolded proteins, potentially leading to accumulation of unfolded proteins that, if released from the endoplasmic reticulum, can have detrimental effects. The accumulation of unfolded proteins in the lumen of the endoplasmic reticulum initiates the UPR. The UPR works to regain endoplasmic reticulum homeostasis by reducing protein translocation into the endoplasmic reticulum, increasing the protein-folding capacity of this organelle, decreasing translation initiation, and increasing protein degradation [28]. Prolonged activation of the UPR leads to apoptosis [29].

In the present study, a heterozygous SNV was detected at chr16:85667696 in *GSE1* gene which results in p.Ala62Thr amino acid change. In previous work, it was demonstrated that engineered candidate cooperating mutations in Gorlin neuroepithelial stem (NES) cells, with mutation of *DDX3X* or loss of GSE1 both accelerated tumorigenesis. These findings demonstrate that human NES cells provide a potent experimental resource for dissecting genetic causation in medulloblastoma [30].

A heterozygous SNV in APC2 gene was encountered at chr19:1457111 which was associated with amino acid change p.Pro359Gln. Continuous activation of the Wnt/β-Catenin signaling has been reported to play an important role in multiple processes of tumor progression, leading to uncontrolled cancer cell proliferation, growth, and survival. miR-1249 targets and suppresses APC2 expression, an important Wnt/β-Catenin pathway-regulated factor. These data suggest that miR-1249 could be a novel therapeutic target for microRNA-mediated cell proliferation in glioma [31]. MUC16 is overexpressed in multiple cancers and plays an important role in tumorigenicity and acquired resistance to therapy. Apart from its protective role in normal physiology, MUC16 contributes to disease progression and metastasis in several malignancies. Identification of neo-antigenic epitopes in MUC16 that correlate with improved survival has raised hopes for developing MUC16-targeted immunotherapy [32].

A heterozygous indel in *MLLT1* gene was recorded in the present study at chr19:6213974; it was associated with amino acid change p.Gln461fs. Interestingly, the *MLLT1* indel was identified in GBM derived p-CSC only. KMT2A (MLL) rearrangements are observed in various types of pediatric and adult leukemia, but only one adult case report has so far shown KMT2A (MLL)-MLLT1 gene rearrangements in blastic plasmacytoid dendritic cell neoplasm (BPDCN) [33].

**Fig. 3:**
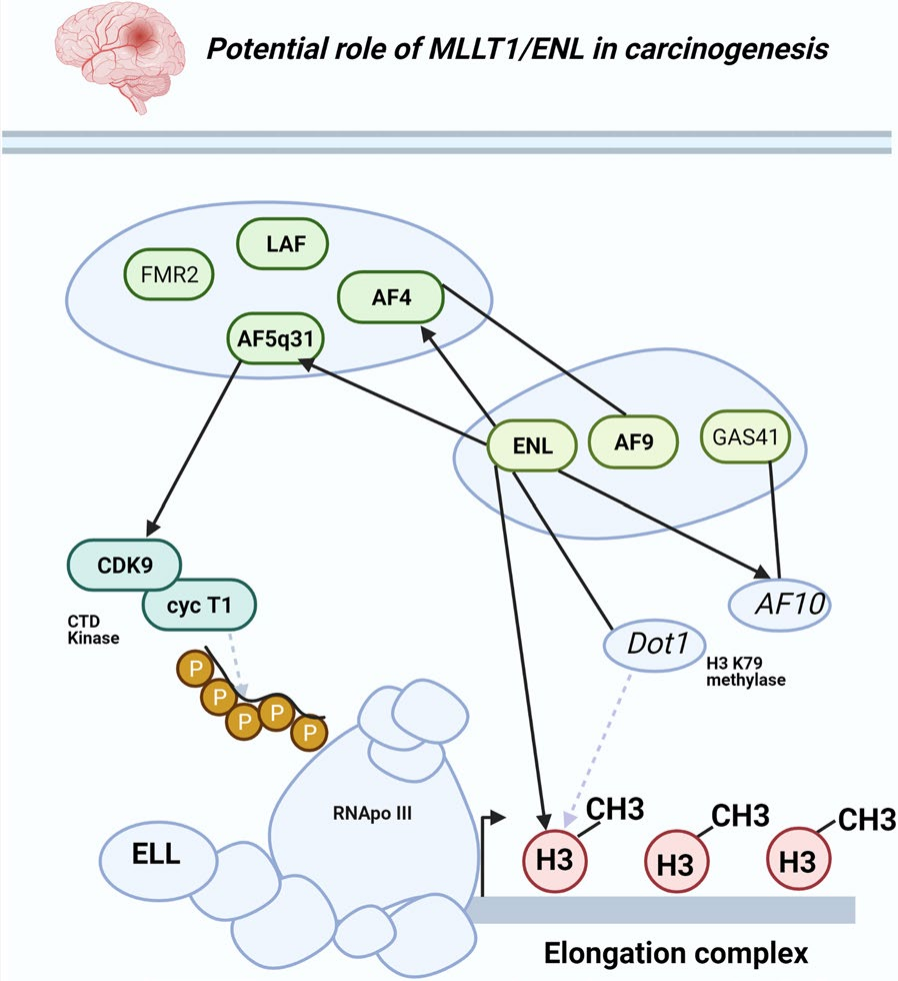
Transcriptional elongation and potential role of nuclear MLL fusion partners. The cartoon show proteins identified in MLL translocations in bold and protein families sharing structural homology are enriched. Protein-protein interactions are indicated by black arrows, and lines. Enzymatic activities that are involved in the transcriptional elongation process are symbolized by dashed grey arrows (Adapted from Zeisig et al [34]

From these findings, we hypothesize that *MLLT1* may be of importance in stem cell differentiation/glioma pathogenesis. However, the implications and potential downstream effects of this genomic variant are not explored. The lack of germline sequencing data makes inferences about somatic SNVs highly concerning. This may necessitate more future studies on higher number of GBM samples, and further experimentation would be needed to highlight potential downstream effects of identified indel in *MLLT1* gene. In order to fill these caveats, we did an extensive search for the potential role of *MLLT1* gene in relation to different malignancies. There were very few data available in previous publication about the implication of this variants in GBM, and potential downstream effects. However, it was demonstrated that mixed lineage leukemia (MLL) fusion proteins are derived from translocations at 11q23 that occur in aggressive subtypes of leukemia, and MLL is joined to different unrelated proteins to form oncogenic transcription factors. Zeisig et al. [34] demonstrated a direct interaction between several nuclear MLL fusion partners and present evidence for a role of these proteins in histone binding. In two-hybrid studies, ENL (the protein product of *MLLT1* gene) interacted with AF4 and AF5q31 as well as with a fragment of AF10. Overlay and pulldown-assays finally showed a specific and YEATS domain-dependent association of ENL with histones H3 and H1. These studies support a common role for nuclear MLL fusion partners in chromatin biology (Fig. 3).

MLL rearrangements are also present in about 10% of other pediatric and adult acute myeloid leukemia (AML) and acute lymphoid leukemia (ALL). These translocations and others occurring in early life are associated with a dismal prognosis compared with adult leukemias carrying the same translocations. This observation suggests that infant and adult leukemias are biologically distinct but the underlying molecular mechanisms for these differences are not understood. Sinha et al. have developed a novel MLL-ENL embryonic leukemia model in mice that can be used to study some aspects of infant leukemia ontogeny [35].

So, now the question is: is ENL fused to MLL in our GBM CSC? Is ENL fused/rearranged to other protein to make a new chimeric protein with aberrant oncogenic functions? We are currently performing more experimentation to prove this new hypothesis for GBM pathogenesis. Debernardi et al. [36] demonstrated that AF10 is involved in 2 distinct chromosomal translocations associated with hematologic malignancy. The chimeric fusion proteins MLL/AF10 and CALM/AF10, resulting from the t(10;11)(p12;q23) and the t(10;11)(p12;q14), respectively, consistently retain the leucine zipper motif of AF10. The leucine zipper interacted with GAS41, a protein previously identified as the product of an amplified gene in a glioblastoma. GAS41 shows significant homology to the Saccharomyces cerevisiae protein ANC1 and to the human MLL fusion partners AF9 and ENL. The interaction was confirmed in vivo [36].

In addition to genetic mutations represented mainly as SNV and indels that have been encountered in the present investigation in key oncogene genes (such as TP73, PDE4DIP, FN1, KMT2C, MUC6, CREB3L1, GSE1, APC2, MUC16, and MLLT1) previous studies have elucidated that GBM was associated with alteration in other key signature oncogenes such as EGFR and PI3K, and that in over 40% of GBM carries one or more nonsynonymous mutation among the chromatin-modifier genes [37]. Alterations in chromatic rearrangement have been described for other types of cancer such as ovarian [38] and renal [39] carcinoma.

Based on the genomic profile of GBM c- and p-CSC that has been confirmed in the present investigation, most of the SNV that were detected in both c- and p-CSC are represented by common variants that have previously been recorded in other types of cancers. To our knowledge, none of the identified homozygous and heterozygous SNV were previously linked to chemotherapeutic drugs used to treat GBM. Moreover, none of the identified variants were previously reported to play a decisive role in the recurrent nature of GBM, an observation that might indicate that the GBM CSC did not play a significant role both in GBM resistance to chemotherapy and its recurrent nature. Nonetheless, the mutation load of the GBM seems to be an integral part of GBM mutanome.

Recently, several studies have been devoted to elucidating potential targeted therapies for GBM, and several such strategies were designed against key GBM oncogenic genes/pathways such as *BRAF* [40] and *FGFR1/FGFR2/FGFR3* [40]. In our previous studies, Notch inhibition significantly impaired cell growth of c-CSC compared to p-CSC, Besides, p-CSC are more refractory to anti-EGFR targeting either alone or in combination with the anti-Notch1 drug compared to c-CSC [3], suggesting that p-CSC possess a different genetic background which confers them resistance to the anti-tumor agents [3]. Simultaneous targeting of EGFR and PDGFR negatively impacted both c-CSC and p-CSC. In another study, the newly discovered PDGFRα/Stat3/Rb1 regulatory axis might represent a potential target for the treatment of refractory p-CSC [4]. We also reported that the interference of Notch1 target Hes1 overcomes the resistance of CSC to GSI-X [2].

The majority of GBM tumors had a complex genome and transcriptome, and usually, they were associated with a high frequency of structural variants on the q arm of chromosome 12, involving the MDM2 and CDK4 genes. This may be a functional alteration relevant to GBM [41]. This view didn’t match the SNV identified in the analysis of the genetic mutations of GBM-associated p- and c-CSC, which might indicate that these key GBM oncogenic genes are mainly relevant to the primary GBM tumor cells rather than to the GBM-associated CSC genes.

Nearly half of GBM tumors display a complex alteration in the EGFR genes as represented by fusion and deletion that compose essential features of the somatic mutations associated with GBM [42]. Despite the main role of EGFR deletion/fusion in the survival and growth maintenance of GBM, other different EGFR alterations might also be encountered. Such alterations might induce variable responses to other targeted therapeutic modalities.

Whether or not the identified GBM genomic alterations are in concordance with the proteomic variations as reflected in the downstream molecular pathways, there is still a need for further investigation, and targeting the altered genomic pathways should be directed not only to key oncogenic genes encountered within the GBM primary tumor cells but also to downstream signaling components along a pathway of GBM-associated CSC.

## Conclusion

The poor prognostic outcome of GBM is attributed to the presence of cancer stem cells (CSC) which confer resistance against standard chemo- and radiotherapeutics modalities. In the present study, potential differences in genetic variants between c-CSC versus p-CSC derived from four GBM patients have been investigated. By comparative analyses, we identified common gene mutations in all GBM c-CSC, and p-CSC: *TP73, PDE4DIP, FN1, KMT2C, MUC6, CREB3L1, GSE1, APC2, MUC16*. A potential deleterious variant was a frameshift deletion at Gln461fs in *MLLT1* gene that was encountered in p-CSC only. Further investigation is required to confirm the presence of the identified mutations in patient tissue samples, as well as the significance of the frameshift mutation in the MLLT1 gene on GBM biology and response to therapy based on genomic functional experiments.

## Supplementary Information

**Additional file 1: Table S1.** Allelic frequency of identified variants.

**Additional file 2: Table S2.** Sample identity-check.

**Additional file 3: Table S3.** Exome sequences data with the summary for the center and periphery-specific variants detected in all patients.

**Additional file 4: Table S4.** Variants detected in all patients, only in the periphery sample.

## Data Availability

We had included all the data and materials in the final version of the manuscript. Bushman Lab Cancer Gene List: http://www.bushmanlab.org/links/genelists. COSMIC: http://cancer.sanger.ac.uk/cosmic
