# Supplementary material for: Exome sequencing of glioblastoma-derived cancer stem cells reveals rare clinically relevant frameshift deletion in MLLT1 gene
Source: Cancer Cell Int. 2022 Jan 7;22:9. doi: 10.1186/s12935-021-02419-4 (PMC8740446; doi:10.1186/s12935-021-02419-4)
Supplement: Supplementary file 1 — Additional file 1: Table S1. Allelic frequency of identified variants. [file 12935_2021_2419_MOESM1_ESM.docx]

| Sample | Internal ID | Barcode | Type | Organism |
| --- | --- | --- | --- | --- |
| c-CSC1 | 1 | 1 | core glioblastoma cancer stem cells | Human |
| p-CSC1 | 2 | 2 | peritumor glioblastoma cancer stem cells | Human |
| c-CSC2 | 3 | 3 | core glioblastoma cancer stem cells | Human |
| p-CSC2 | 4 | 4 | peritumor glioblastoma cancer stem cells | Human |
| c-CSC3 | 5 | 5 | core glioblastoma cancer stem cells | Human |
| p-CSC3 | 6 | 6 | peritumor glioblastoma cancer stem cells | Human |
| c-CSC4 | 7 | 7 | core glioblastoma cancer stem cells | Human |
| p-CSC4 | 8 | 8 | peritumor glioblastoma cancer stem cells | Human |

**Table 1 – a list of analyzed samples**
