# Supplementary material for: Exome sequencing of glioblastoma-derived cancer stem cells reveals rare clinically relevant frameshift deletion in MLLT1 gene
Source: Cancer Cell Int. 2022 Jan 7;22:9. doi: 10.1186/s12935-021-02419-4 (PMC8740446; doi:10.1186/s12935-021-02419-4)
Supplement: Supplementary file 2 — Additional file 2: Table S2. Sample identity-check. [file 12935_2021_2419_MOESM2_ESM.docx]

| Sample | Total reads | Mapped reads | Mapping rate (%) | On target (%) |
| --- | --- | --- | --- | --- |
| c-CSC1 | 12,563,290 | 12,520,354 | 99.66% | 94.76% |
| p-CSC1 | 21,381,239 | 21,313,206 | 99.68% | 96.03% |
| c-CSC2 | 23,975,039 | 23,897,330 | 99.68% | 95.27% |
| p-CSC2 | 23,223,428 | 23,154,328 | 99.70% | 95.68% |
| c-CSC3 | 29,635,293 | 29,533,507 | 99.66% | 95.32% |
| p-CSC3 | 20,265,457 | 20,192,375 | 99.64% | 95.85% |
| c-CSC4 | 23,921,320 | 23,823,634 | 99.59% | 95.27% |
| p-CSC4 | 18,706,321 | 14,175,267 | 75.78% | 87.85% |

**Table 2 –** Mapping statistics
