# Supplementary material for: Exome sequencing of glioblastoma-derived cancer stem cells reveals rare clinically relevant frameshift deletion in MLLT1 gene
Source: Cancer Cell Int. 2022 Jan 7;22:9. doi: 10.1186/s12935-021-02419-4 (PMC8740446; doi:10.1186/s12935-021-02419-4)
Supplement: Supplementary file 3 — Additional file 3: Table S3. Exome sequences data with the summary for the center and periphery-specific variants detected in all patients. [file 12935_2021_2419_MOESM3_ESM.docx]

|  | Reference | Variant | Type | Zygosity | Amino acid change | Oncogene |
| --- | --- | --- | --- | --- | --- | --- |
| chr1:3644245 | G | T | SNV | HET | p.Gly299Val | TP73 |
| chr1:144866643 | G | A | SNV | HET | p.Arg1867Cys | PDE4DIP |
| chr1:144879375 | T | C | SNV | HET | p.Lys1359Glu | PDE4DIP |
| chr1:144882823 | C | T | SNV | HET | p.Ala1066Thr | PDE4DIP |
| chr1:144917841 | T | C | SNV | HET | p.His482Arg | PDE4DIP |
| chr1:144918957 | T | A | SNV | HET | p.Glu410Val | PDE4DIP |
| chr1:144922583 | G | A | SNV | HET | p.Ser275Leu | PDE4DIP |
| chr1:144994658 | C | A | SNV | HET | p.Arg25Leu | PDE4DIP |
| chr2:216272900 | T | G | SNV | **HOM** | p.Thr817Pro | FN1 |
| chr7:151945071 | G | GT | INDEL | HET | p.Tyr816Ter | KMT2C |
| chr11:1017183 | G | T | SNV | HET | p.Pro1873Gln | MUC6 |
| chr11:1017220 | T | C | SNV | HET | p.Thr1861Ala | MUC6 |
| chr11:1017325 | A | C | SNV | HET | p.Tyr1826Asp | MUC6 |
| chr11:1017337 | TC | CA | MNV | HET | p.Gln1821_Thr1822delinsHisAla | MUC6 |
| chr11:46342081 | TG | T | INDEL | **HOM** | splicesite_3 | CREB3L1 |
| chr11:46342259 | A | AG | INDEL | **HOM** | splicesite_5 | CREB3L1 |
| chr16:85667696 | G | A | SNV | HET | p.Ala62Thr | GSE1 |
| chr19:1457111 | C | A | SNV | HET | p.Pro359Gln | APC2 |
| chr19:9087615 | T | A | SNV | **HOM** | p.Lys1400Asn | MUC16 |

**Table 3 –** Variants detected in all patients, both in the centre and the periphery sample
