# Supplementary material for: Exome sequencing of glioblastoma-derived cancer stem cells reveals rare clinically relevant frameshift deletion in MLLT1 gene
Source: Cancer Cell Int. 2022 Jan 7;22:9. doi: 10.1186/s12935-021-02419-4 (PMC8740446; doi:10.1186/s12935-021-02419-4)
Supplement: Supplementary file 4 — Additional file 4: Table S4. Variants detected in all patients, only in the periphery sample. [file 12935_2021_2419_MOESM4_ESM.docx]

| Position | Reference | Variant | Type | Zygosity | Amino acid change | Oncogene |
| --- | --- | --- | --- | --- | --- | --- |
| chr19:6213974 | CTG | CT | INDEL | HET | p.Gln461fs | MLLT1 |

**Table 4 –** Variants detected in all patients, only in the periphery sample
